# Aggressive cutaneous squamous cell carcinoma in a patient on Janus-kinase inhibitor therapy

**DOI:** 10.1016/j.jdcr.2024.11.042

**Published:** 2025-01-04

**Authors:** Ana M. Aragon Sierra, Kevin J. Donohoe, Daniel J. Canaday, Ernest D. Gomez, Scharukh M. Jalisi, Matthew M. Koomey, Victor M. Aviles, Kenneth K. Yu

**Affiliations:** a Cambridge Health Alliance, Cambridge, Massachusetts; b Harvard Medical School, Boston, Massachusetts; c Beth Israel Deaconess Medical Center, Boston, Massachusetts; d Beth Israel Deaconess Hospital – Plymouth, Plymouth, Massachusetts; e Hematology Oncology Specialists of Cape Cod, North Falmouth, Massachusetts; f Plymouth Dermatology Associates, Plymouth, Massachusetts

**Keywords:** cemiplimab, cutaneous malignancy, cutaneous squamous cell carcinoma, immunosuppression, immunotherapy, JAK inhibitor, Jakafi, JAKi, Janus-kinase inhibitor, malignancy risk, malignancy, pembrolizumab, ruxolitinib, squamous cell carcinoma

## Introduction

The risk of skin cancer in immunosuppressed patients is well known; however, the risk posed by newer immunosuppressants (often marketed as “immunomodulators”), such as the Janus-kinase inhibitor (JAKi), ruxolitinib, may be underestimated and underreported.^1^, ^2^, ^3^ We report a case of aggressive, multiply recurrent, cutaneous squamous cell carcinoma (SCC), resistant to multiple treatments, including immunotherapy. Strikingly, the disease responded to immunotherapy only after discontinuing ruxolitinib, implicating the profound immunosuppression induced by chronic systemic JAKi therapy in treatment resistance. Food and Drug Administration-approved for adults with myelofibrosis and polycythemia vera, ruxolitinib is a JAKi currently being used to treat various inflammatory skin conditions, including psoriasis, atopic dermatitis, vitiligo, and lupus.^3^ This case highlights the risks of aggressive cutaneous malignancy and profound immunosuppression in patients on chronic systemic JAKis, warranting a cautious approach to their use.

## Case Report

**Fig 1:**
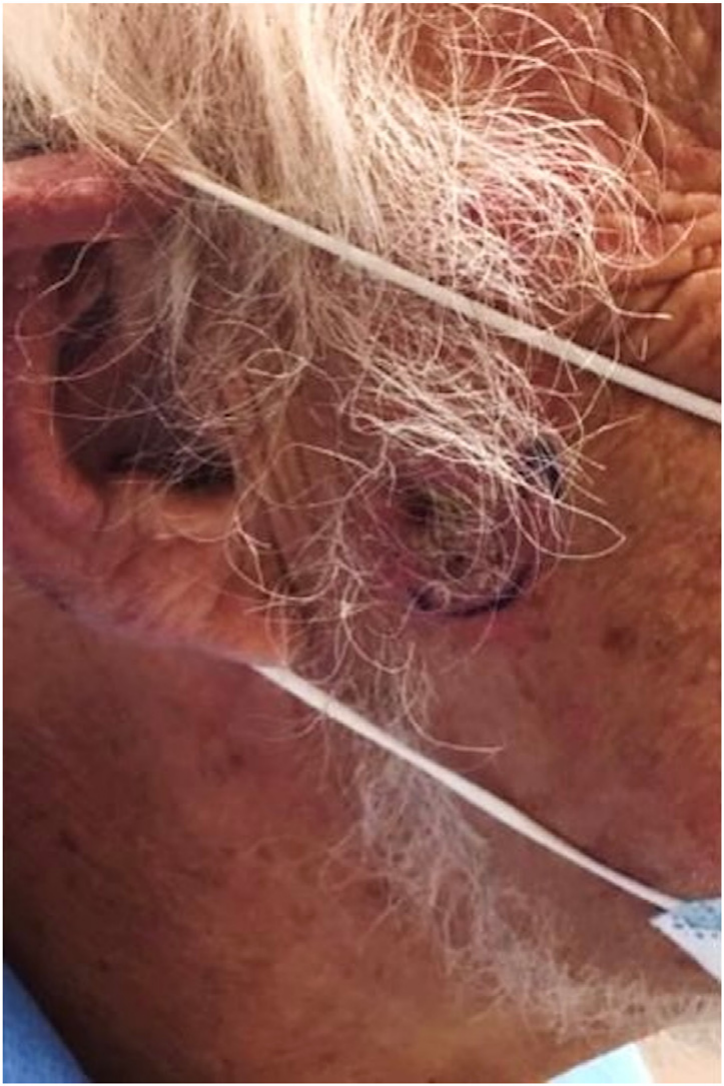
Initial presentation of squamous cell carcinoma before first Mohs resection.

A 72-year-old man was seen in a cutaneous oncology clinic for a multiply recurrent SCC on the right preauricular cheek. He had a decades-long history of polycythemia vera, refractory to hydroxyurea and phlebotomy; he had been on ruxolitinib 20 mg twice a day orally for 6 years. The SCC (Fig 1) first presented on the right cheek 1 year ago as a local, well-differentiated SCC (AJCC8 stage T1N0M0, Clinical stage IA, and Brigham Women’s Hospital stage T1), and was treated with Mohs micrographic surgery with reportedly clear margins. However, the SCC recurred 7 months postoperatively, and was again treated with Mohs micrographic surgery. No postoperative adjuvant radiation was given. Four months later, a deep bleeding nodule was noted within the surgical scar when the patient presented to the cutaneous oncology clinic for an unrelated SCC. Biopsy showed a moderately-differentiated SCC, consistent with a second recurrence.

Staging computed tomography (CT) of the head and neck revealed a poorly defined dermal and subcutaneous mass measuring 1.9 × 1.0 × 1.7 cm in the right preauricular region. Tumor invasion of adjacent vessels and the parotid duct could not be excluded, with extension toward the right masseter muscle (T2N0M0, stage IB). Neoadjuvant immunotherapy with cemiplimab was initiated after Multidisciplinary Tumor Board review, in an effort to downstage the tumor to improve resectability and minimize surgical morbidity. Restaging magnetic resonance imaging after 2 cycles of cemiplimab showed tumor progression, now measuring 2.5 × 4.5 × 4.6 cm, with invasion into the right masseter muscle and involvement of facial nerve branches. Positron emission tomography-CT (PET-CT) showed no distant metastases (T3N0M0, stage IIA).

The ruxolitinib dose was reduced to 20 mg every other day. The patient underwent definitive surgical resection by Head and Neck Oncology, consisting of radical parotidectomy with sacrifice of the facial nerve and right side of the neck lymph node dissection, followed by free flap reconstruction. Permanent section margins and nodes were negative, but the flap failed because of postoperative infection, requiring further facial plastic reconstruction, delaying adjuvant radiation therapy. During this period, the ruxolitinib schedule was adjusted to 10 mg daily. Adjuvant radiation was completed 3 months after the definitive resection. Three months later, restaging PET-CT scans were free of metastases, and no signs of recurrence were found on physical examination.

**Fig 2:**
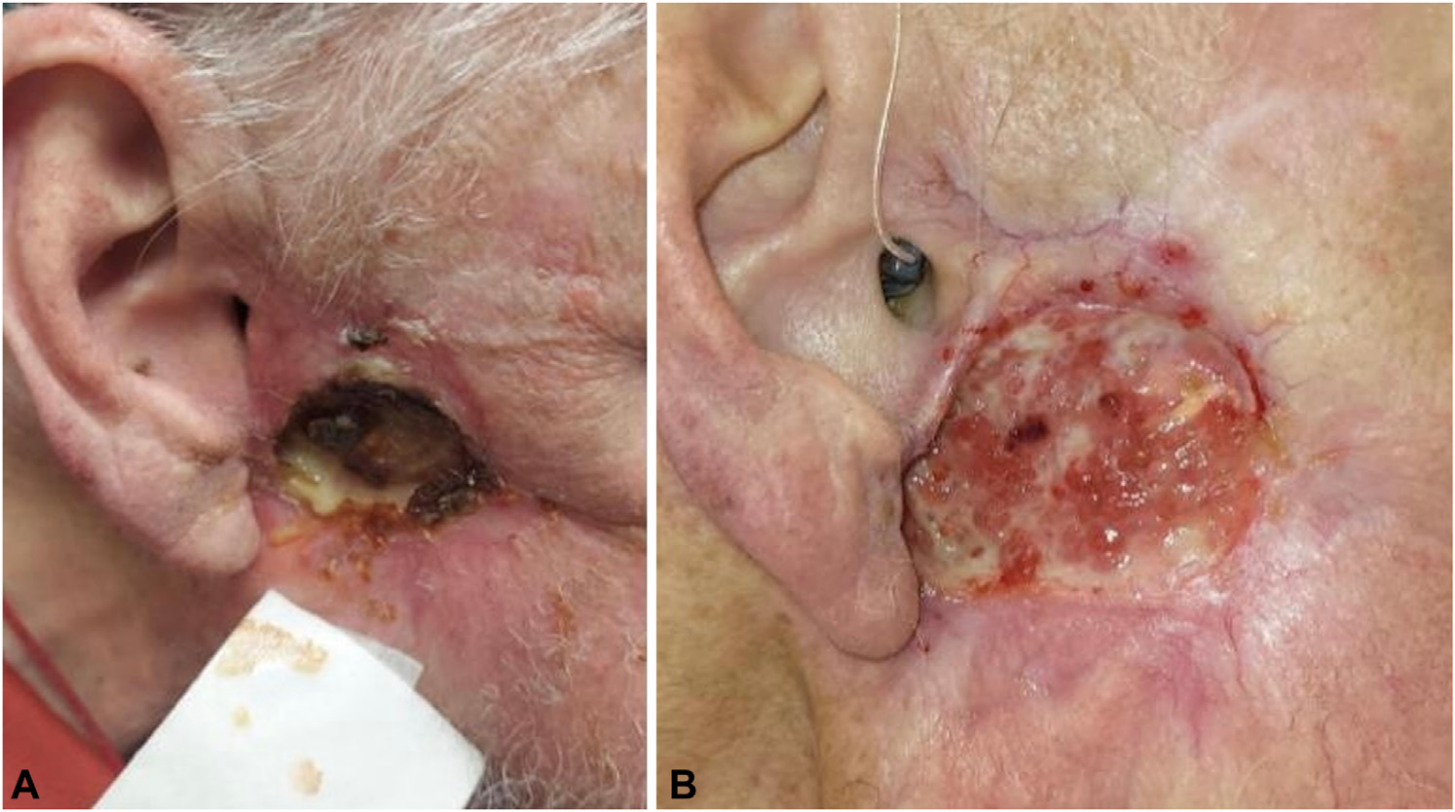
**A,** Image of SCC before ruxolitinib taper and starting pembrolizumab treatment. **B,** Image of SCC 1 month after ruxolitinib was stopped and after 3 doses of pembrolizumab, with pink healthy granulation tissue in the wound bed. *SCC*, Squamous cell carcinoma.

**Fig 3:**
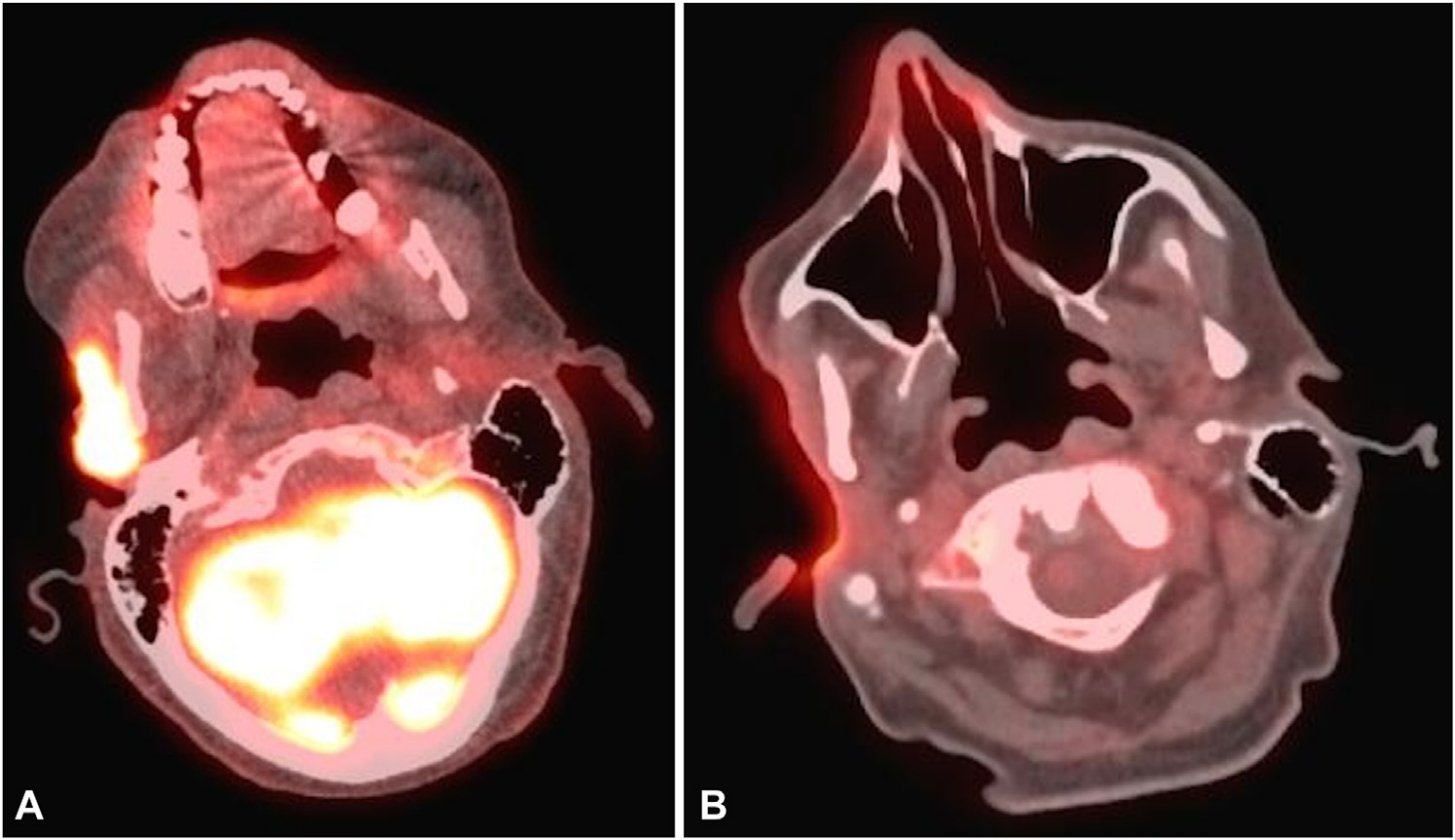
**A,** PET-CT of head before ruxolitinib taper and starting pembrolizumab treatment. **B,** PET-CT of head 2 months after ruxolitinib was stopped and after 4 doses of pembrolizumab. *PET-CT*, Positron emission tomography-computed tomography.

However, 2 months later, an ulcer developed within the operative site (Fig 2, *A*). Biopsy showed invasive moderately-differentiated SCC within a keloidal scar with extension into surrounding soft tissue (T4aN0M0 stage III). Tumor Board initially recommended further surgical resection, with adjuvant immunotherapy and radiation, and transitioning from ruxolitinib to interferon alfa for his polycythemia vera. However, the tumor progressed rapidly, and a preoperative magnetic resonance imaging of the neck showed a 4.5 × 2.7 × 4.7 cm mass abutting the right mandibular ramus and right mastoid air cells, with invasion into the right masseter muscle. PET-CT (Fig 3, *A*) revealed increased fluorodeoxyglucose uptake in the right cheek lesion (maximum standardized uptake value of 9.5) and numerous new pulmonary nodules concerning for metastases, confirmed on chest CT (T4aN1M1 stage IV); surgery was canceled.

The patient was started on interferon-alpha therapy for his polycythemia vera, and a 1-month ruxolitinib taper began. Interestingly, as ruxolitinib was tapered, other contemporaneous squamous skin lesions also appeared to regress or completely disappear. After ruxolitinib was stopped, immunotherapy with pembrolizumab was initiated under compassionate use. After the third dose of pembrolizumab, the cheek ulcer began to granulate and clinically stabilize (Fig 2, *B*). A restaging PET-CT (Fig 3, *B*), 3 weeks after the fourth dose of pembrolizumab, revealed complete resolution of the pulmonary nodules and fluorodeoxyglucose avidity, and decrease in fluorodeoxyglucose uptake in the primary site in the right cheek. However, the patient’s course was complicated by severe immunotherapy-related oral mucositis. He was admitted to a local hospital for nutritional support and systemic corticosteroids. Despite initial improvement in his condition, he developed pneumonia while in the hospital, and his clinical course took a precipitous turn. He was placed on hospice care, and expired shortly after.

## Discussion

This case of an aggressive, refractory SCC that became responsive to immunotherapy only after discontinuation of ruxolitinib, highlights the cutaneous malignancy risks and implicates the profound immunosuppression of JAKi therapy in the tumor’s aggressive behavior.^4^ Recent studies have demonstrated that JAKis may be associated with an increased risk of cutaneous malignancies.^5^, ^6^, ^7^, ^8^ The emergence of the patient’s aggressive tumor 6 years into JAKi treatment appears to parallel the trajectory of cutaneous malignancies associated with azathioprine therapy.^6^ A study analyzing US Food and Drug Administration adverse event data showed that JAKis are associated with malignant skin tumors, with ruxolitinib being the riskiest.^4^

Aggressive skin cancer development may not become evident until several years after the initiation of JAKi therapy. Transplant patients, with similarly profound immunosuppression, also provide a precedent for the aggressive behavior of SCC under such conditions.^9^ An analysis of global safety data supports increased risks of skin cancers in patients on JAKis, underscoring the need for vigilant screening and risk management in this patient population.^10^ The lack of long-term cutaneous malignancy data on chronic use of the newer systemic JAKis warrants caution in their application. Acknowledging these risks, implementing proactive screening, and developing strategies to minimize chronic immunosuppression (through drug holidays or pulse-dose therapy) will help clinicians more safely make use of this class of powerful immunosuppressants.

## Conflicts of interest

None disclosed.
